# Comparative Single Vesicle Analysis of Aqueous Humor Extracellular Vesicles before and after Radiation in Uveal Melanoma Eyes

**DOI:** 10.3390/ijms25116035

**Published:** 2024-05-30

**Authors:** Shreya Sirivolu, Chen-Ching Peng, Paolo Neviani, Benjamin Y. Xu, Jesse L. Berry, Liya Xu

**Affiliations:** 1 The Vision Center at Children’s Hospital Los Angeles, Los Angeles, CA 90027, USA; sirivolu@usc.edu (S.S.); ppeng@chla.usc.edu (C.-C.P.); 2 USC Roski Eye Institute, Keck School of Medicine, University of Southern California, Los Angeles, CA 90033, USA; 3 Extracellular Vesicle Core, Children’s Hospital Los Angeles, Los Angeles, CA 90027, USA; 4 The Saban Research Institute, Children’s Hospital Los Angeles, Los Angeles, CA 90027, USA; 5 Norris Comprehensive Cancer Center, Keck School of Medicine, University of Southern California, Los Angeles, CA 90033, USA

**Keywords:** extracellular vesicles (EVs), uveal melanoma, aqueous humor (AH), liquid biopsy, ocular cancer

## Abstract

Small extracellular vesicles (sEVs) have been shown to promote tumorigenesis, treatment resistance, and metastasis in multiple cancer types; however, sEVs in the aqueous humor (AH) of uveal melanoma (UM) patients have never previously been profiled. In this study, we used single particle analysis to characterize sEV subpopulations in the AH of UM patients by quantifying their size, concentration, and phenotypes based on cell surface markers, specifically the tetraspanin co-expression patterns of CD9, CD63, and CD81. sEVs were analyzed from paired pre- and post-treatment (brachytherapy, a form of radiation) AH samples collected from 19 UM patients. In post-brachytherapy samples, two subpopulations, CD63/81+ and CD9/63/81+ sEVs, were significantly increased. These trends existed even when stratified by tumor location and GEP class 1 and class 2 (albeit not significant for GEP class 2). In this initial report of single vesicle profiling of sEVs in the AH of UM patients, we demonstrated that sEVs can be detected in the AH. We further identified two subpopulations that were increased post-brachytherapy, which may suggest radiation-induced release of these particles, potentially from tumor cells. Further study of the cargo carried by these sEV subpopulations may uncover important biomarkers and insights into tumorigenesis for UM.

## 1. Introduction

Extracellular vesicles (EVs) are the collective term for various secreted membrane-enclosed nano-sized vesicles released by virtually every cell type [1]. EVs are classified by size, with small EVs (sEVs), ranging from 30 to 150 nm, being the predominant size found in intraocular biofluids [2]. Among EVs, sEVs are of the greatest interest as potential cancer biomarkers [3,4] with tumor-derived sEVs being shown to promote tumor formation, progression, resistance, immune response regulation, and metastases [4,5,6,7], resulting in the emerging role of sEVs in biomarker research [8]. EVs have also been reported for different malignances, such as colorectal cancer, to carry oncogenic factors that can trigger malignant transformation in target cells [9]. The surfaces of sEVs are highly enriched in tetraspanin, a protein superfamily that organize membrane microdomains by forming clusters and interacting with a large variety of transmembrane and cytosolic signaling proteins [10,11,12,13]. While they have been used for the phenotypic expression profiling of EVs, tetraspanins have a role beyond serving as cell surface markers that includes extracellular vesicle biogenesis, cargo selection, cell targeting, and cell uptake [14].

We previously published that a distinct eye-specific subpopulation of sEVs can be detected in pediatric aqueous humor (AH) in patients with several ocular diseases, which included congenital cataract, congenital glaucoma, pediatric retinal disease, and retinoblastoma [15]. We identified enrichment of the mono-CD63+ sEV subpopulation in the AH across all disease types [15]. In a study analyzing EVs in multiple cancers, CD63+ vesicles were not present in human plasma, serum, and bone marrow and were present in 10% or fewer samples of lymphatic and bile duct fluid [16]. This suggests that the mono-CD63+ sEV subpopulation may be specific to the eye and detectable in the AH. The specific role of CD63+ sEVs in ocular tumors is yet to be described. Previous reports suggest that CD63 is closely associated with lysosomal trafficking and is a key player in exosome formation and release by participating in the endosomal sorting complex required for transport (ESCART)-independent pathway [17]. In studying patients with retinoblastoma, a primary pediatric ocular malignancy, we observed a significantly dominant subpopulation of CD63/81+ sEVs [18]. This subpopulation was more enriched before treatment and in patients with more significant tumor burden, suggesting they are a tumor-derived subpopulation.

The composition of EVs in the AH of uveal melanoma (UM) patients has not been previously reported. Uveal melanoma is the most common primary ocular malignancy in adults [19]. It is a relatively rare disease (incidence: 5.1 cases per million per year), with tumors located in either the iris (4%), ciliary body (6%), or the choroid (90%) [19]. Plaque brachytherapy, a form of localized radiation therapy, is a standard-of-care procedure to preserve the eye in uveal melanoma patients. The procedure involves the placement of a radioactive plaque onto the scleral wall, followed by its subsequent removal several days later [20]. Gene expression profiling (GEP) is a widely used prospectively validated tool to stratify the risk of metastasis by assigning UM patients to two highly prognostic molecular classes: class 1 (low metastatic risk) and class 2 (high metastatic risk) [21]. Our aim is to use single particle analysis to characterize sEV subpopulations in the AH of UM patients by quantifying their size, concentration, and phenotypes based on tetraspanin expression patterns. Our analysis included paired pre- and post-brachytherapy AH samples, which were further stratified by GEP class and by tumor location.

## 2. Results

### 2.1. Patient Clinical Characteristics and Demographics

Nineteen UM patients were included in this study, with paired AH samples (pre- and post-brachytherapy collected from each patient). Based on a clinically validated and widely used 15-gene expression profile test performed on tumor biopsy samples by Castle Biosciences, 12 patients were GEP class 1, 4 patients were GEP class 2, and 3 patients had unknown GEP classification. In this study, posterior tumors are defined as choroidal tumors that do not involve the iris or ciliary body. Anterior tumors are defined as tumors that involve the iris and/or ciliary body. A total of 11 patients had posterior tumors, and 8 patients had anterior tumors. When patients were grouped by either GEP classification (Table A1) or tumor location (Table A2), the AJCC (American Joint Committee on Cancer) stage showed statistically significant differences between groups. No patients withdrew or were lost to follow-up over the study period. Aqueous humor samples from five glaucoma (GLC) patients taken at the time of routine cataract surgery were included as the non-tumor control group.

### 2.2. Small-Extracellular-Vesicle Size and Concentration Profiling

**Figure A1:**
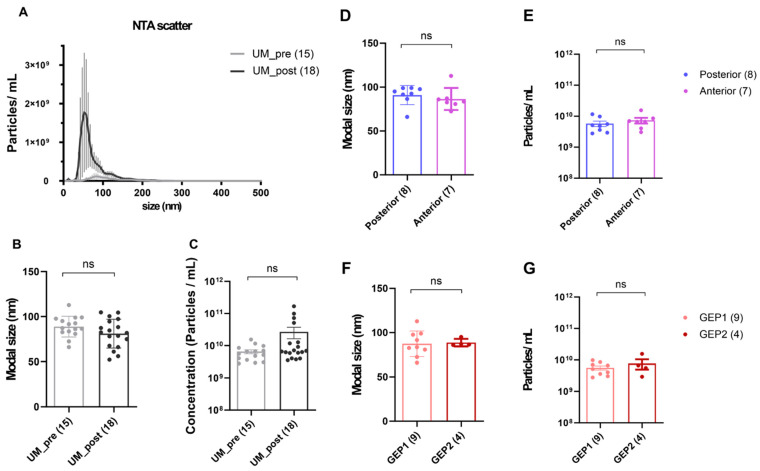
Characterization of EVPs from unprocessed AH by Nanoparticle Tracking Analysis. (**A**–**C**) Average particle counts versus size distribution, average modal size, and average particle concentration in pre- and post-brachytherapy samples. (**D**,**E**) Average modal size and particle concentration in anterior and posterior tumors. (**F**,**G**) Average modal size and particle concentration in GEP class 1 and GEP class 2 tumors. Error bars represent standard deviations obtained from each study group. Due to AH availability, full experimental cohort was not included in this analysis.

Unprocessed AH was used for extracellular-vesicle and -particle (EVP) size and concentration profiling via Nanoparticle Tracking Analysis (NTA), with results shown in Figure A1. Due to sample availability, 15 pre-brachytherapy AH samples and 18 post-brachytherapy AH samples were used for this analysis. All nanoparticles’ modal size was <150 mm, suggesting that sEVs (which range between 30 and 150 nm) are the major EV constituent in AH. The results demonstrate no significant difference in average particle counts per size, average modal size, or average concentration between pre- and post-brachytherapy samples (Figure A1A–C). Average modal size and particle concentration also did not demonstrate any significant differences in tumor location (Figure A1D,E) or GEP classes (Figure A1F,G) in pre-brachytherapy samples.

### 2.3. Tetraspanin Expression Profiling and Quantification

**Figure A2:**
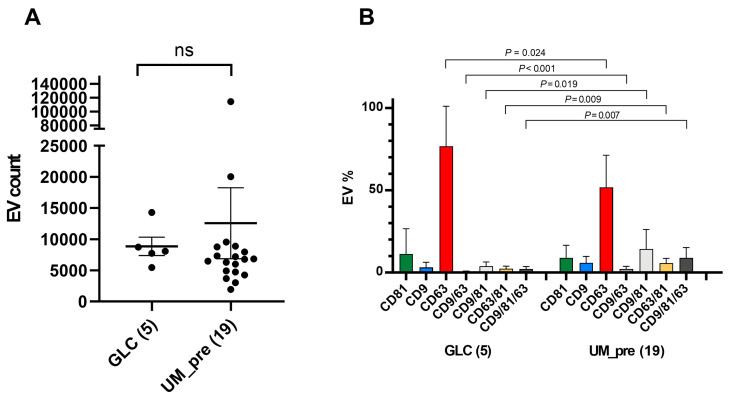
Quantitative comparison of sEV tetraspanin subpopulation expression profiles in glaucoma (GLC) and UM pre-brachytherapy (UM_pre) AH samples. (**A**) Mean total sEV counts compared between GLC and UM_pre AH samples. (**B**) Mean sEV subpopulation percentages compared between GLC and UM_pre AH samples.

Total sEV counts and tetraspanin-based subpopulation profiles between the non-tumor control group and the UM group are shown in Figure A2. Total sEV counts between 5 glaucoma (GLC) and the 19 pre-brachytherapy UM samples showed no significant difference. CD63+ sEVs was the dominant subpopulation in both GLC samples and UM samples, with GLC having a significantly higher percentage. UM pre-brachytherapy samples exhibited a more diverse sEV subpopulation profile than GLC, with significantly increased percentages of CD9/63+, CD9/81+, CD63/81+, and CD9/81/63+ sEVs.

#### 2.3.1. Comparison of Small Extracellular Vesicle Profiles between Paired Pre- vs. Post-Brachytherapy AH Samples

**Figure 1:**
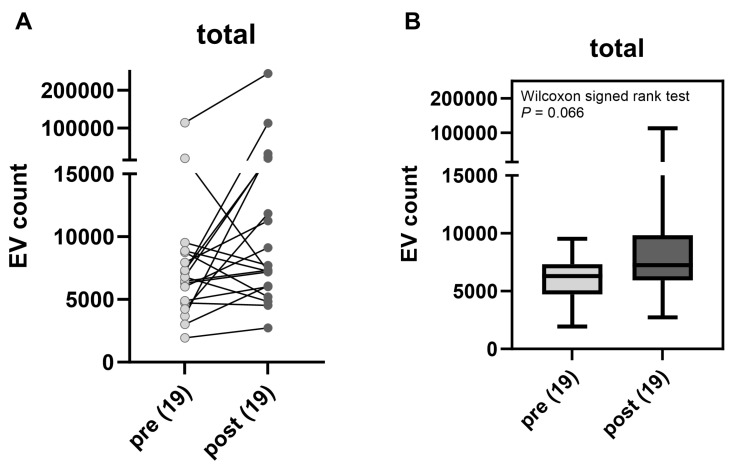
Comparison of total sEV counts pre- and post-brachytherapy. (**A**) Paired sEV counts for each sample. (**B**) Box-and-whisker plot of pooled sEV counts.

sEV counts are shown between the 19 paired pre- and post-brachytherapy AH samples in Figure 1. An increasing trend was observed for the total EV counts after therapy.

**Figure 2:**
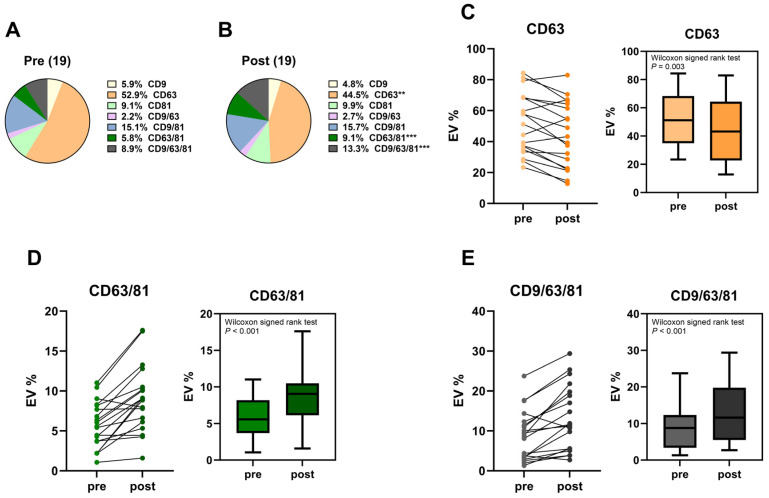
sEV subpopulation dynamics. (**A**,**B**) Comparison of percent composition of sEV subpopulations pre- and post-brachytherapy. Percentages of CD63+ (**C**), CD63/81+ (**D**), and CD9/63/81+ (**E**). sEV subpopulations pre- and post-brachytherapy, shown as paired percentage for each sample and pooled percentage for all samples. ** *p* < 0.01, *** *p* < 0.001.

Figure 2 illustrates the changes in the percent composition of sEV subpopulations identified by expression profiles by using three tetraspanin markers (CD9+, CD63+, and CD81+). In comparison to pre-brachytherapy samples (Figure 2A), the mean percentage of CD63+ sEVs showed a significant decrease in post-brachytherapy samples (*p* = 0.003) (Figure 2A,B). On the other hand, the mean percentages of CD63/81+ sEVs and CD9/63/81+ sEVs showed a significant increase in post-brachytherapy samples (*p* < 0.001) (Figure 2A,B). Both paired pre- and post-brachytherapy results from each individual sample as well as pooled results for each of these three subpopulations demonstrate these trends (Figure 2C–E).

#### 2.3.2. Comparison of Small Extracellular Vesicle Profiles among Aqueous Humor Samples Stratified by Gene Expression Profile Class and Tumor Location

**Figure 3:**
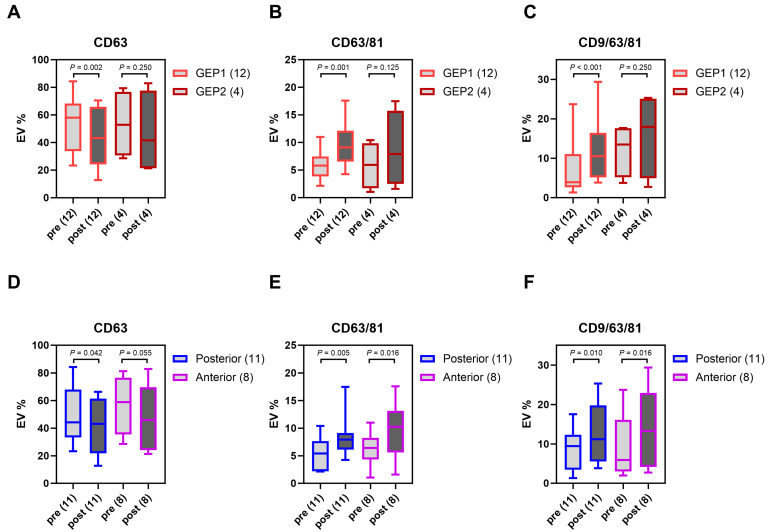
Comparison of percent composition of CD63+, CD63/81+, and CD9/63/81+ sEV subpopulations pre- and post-brachytherapy after GEP class stratification (**A**–**C**) and tumor location stratification (**D**–**F**).

**Figure A3:**
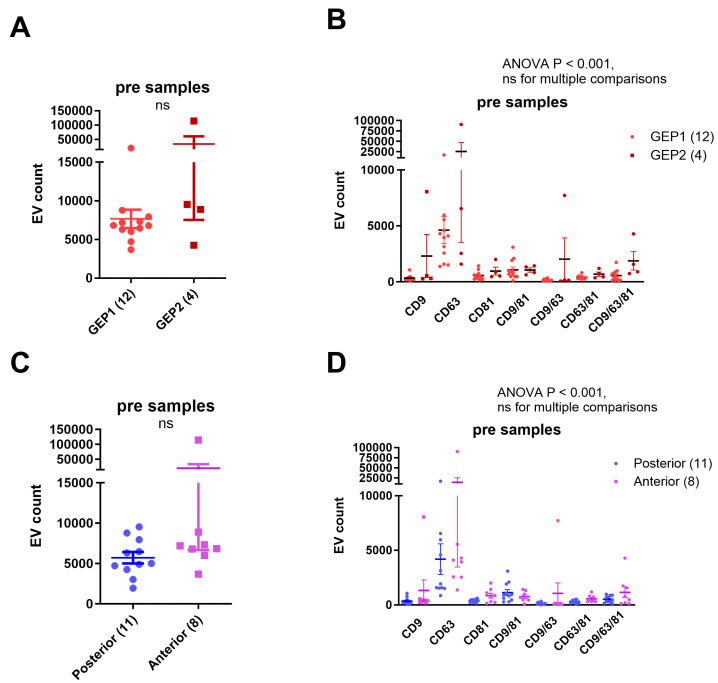
Comparison of pre-brachytherapy AH samples stratified into GEP class and tumor location. (**A**) Total EV counts in GEP class 1 and GEP class 2 tumors. (**B**) sEV tetraspanin expression profiles in GEP class 1 and GEP class 2 tumors. (**C**) Total EV counts in anterior and posterior tumors. (**D**) sEV tetraspanin expression profiles in anterior and posterior tumors.

sEV tetraspanin expression profiles were compared in pre- and post-brachytherapy samples after stratifying by GEP class and tumor location. In pre-brachytherapy samples, there was no significant difference in total sEV counts or sEV subpopulation percentages between GEP classes and between anterior and posterior tumors (Figure A3). The subpopulation percentages of CD63+, CD63/81+, and CD9/63/81+ sEVs were compared for each GEP class between pre- and post-brachytherapy samples, as shown in Figure 3A–C. In GEP class 1 tumors, the mean percentages showed a statistically significant decrease from pre- to post-brachytherapy in CD63+ sEVs (*p* = 0.002) and a statistically significant increase in CD63/81+ sEVs (*p* = 0.001) and CD9/63/81+ sEVs (*p* < 0.001). GEP class 2 samples showed these same trends; however, the results were not significant. The subpopulation percentages of CD63+, CD63/81+, and CD9/63/81+ sEVs were compared for each tumor location between pre- and post-brachytherapy samples, as shown in Figure 3D–F. In posterior tumors, the mean percentages showed a statistically significant decrease from pre- to post-brachytherapy in CD63+ sEVs (*p* = 0.042) and a statistically significant increase in CD63/81+ sEVs (*p* = 0.005) and CD9/63/81+ sEVs (*p* = 0.010). In anterior tumors, the mean percentages showed a statistically significant increase in CD63/81+ sEVs (*p* = 0.016) and CD9/63/81+ sEVs (*p* = 0.016). There was a decrease in the mean percentage of CD63+ sEVs; however, it was not significant.

## 3. Discussion

Herein, we present the first investigation into sEVs in UM patients with the use of single vesicle analysis. We demonstrate that sEVs can be detected in the AH of UM patients and their tetraspanin expression can be profiled by using single vesicle analysis. By comparing paired pre- and post- brachytherapy AH samples from 19 UM patients, the effect of radiation on sEV subpopulations was trended in all samples and further analyzed after sample stratification into GEP class and tumor location.

We have previously shown in UM patients that post-brachytherapy AH had significantly higher DNA and miRNA concentrations than pre-brachytherapy AH [22]. Higher tumor-derived nucleic acids have also been shown in plasma. In Francis et al., patients undergoing 3-day plaque brachytherapy had significantly more tumor-derived cell-free DNA in the plasma 48–72 h after plaque brachytherapy compared with less than 48 h after therapy. It was speculated in both studies that radiation from brachytherapy caused tumor cell necrosis and lysis, releasing DNA into the AH and blood [23].

Our results in this current study suggest that brachytherapy can similarly result in the release of sEVs into the AH. Two separate platforms, NTA NanoSight NS300 and ExoView R100, yielded consistent results of clear increasing trends in total sEV counts and concentrations in post-brachytherapy samples. While the results are consistent in showing an increase in sEVs post-brachytherapy, a limited sample size and high sample variability may be the cause of the statistical results not being significant. Two subpopulations, CD63/81+ and CD9/63/81+ sEVs, demonstrated significantly increased percentages in post-brachytherapy samples, suggesting radiation-induced release of these vesicles (Figure 2). The increase in percentage of these two subpopulations is presumed to be the cause of the decrease in the percentage of the normally dominant CD63+ subpopulation, an AH-specific subpopulation reported in several pediatric ocular disease states [15].

When stratified based on GEP class, significantly increased percentages of CD63/81+ and CD9/63/81+ sEV subpopulations in post-brachytherapy samples were seen in GEP class 1 (low metastatic risk) tumors. While these same trends existed in GEP class 2 (high metastatic risk) tumors, the increase was not significant. Studies investigating differences in treatment response to brachytherapy between both GEP classes have not reached a consistent result. Some studies revealed that GEP class 1 tumors regress more rapidly [24,25], while others showed that GEP class 2 tumors had more rapid regression [26] or no statistically significant difference [27,28]. For this reason, we cannot conclude that inherent differences in responses to radiation between both classes of tumors result in more sEV release in GEP class 1 tumors. It is more likely that we could not achieve significance in GEP class 2 tumors due to a low sample size (12 GEP 1 pairs vs. 4 GEP 2 pairs). Tumor location does not influence the preferential release of these sEV subpopulations; the percentages of CD63/81+ and CD9/63/81+ sEVs demonstrated a significant increase post-brachytherapy in both anterior and posterior tumors (12 anterior vs. 7 posterior pairs).

The enrichment of CD63/81+ and CD9/63/81+ sEVs in post-brachytherapy samples suggests that these subpopulations may be tumor-derived. However, definitive tumor origin is not evidenced in this study, as these vesicles may have been the result of a radiation-induced change in the surrounding normal cells, such as brachytherapy-induced tissue necrosis resulting in sEV release.

In retinoblastoma eyes, the CD63/81+ sEV subpopulation was also hypothesized to be tumor-derived [18]. Given its association with both UM and retinoblastoma, further investigation into the cargo of these sEVs is warranted. Using UM cell lines, Tsering et al. showed that protein cargo derived from EVs may be involved in tumorigenesis and metastatic dissemination [7]. These studies demonstrate that investigating the cargo of tumor-derived EVs may increase our understanding of critical molecular processes of the tumor. These findings may have direct clinical benefits in the form of biomarkers of disease and drug targeting.

In conclusion, we present the phenotypic profiles of sEV subpopulations collected from AH pre- and post-brachytherapy in uveal melanoma patients. To our knowledge, this is the first time sEVs are reported from UM AH samples. Through the profiling of tetraspanin cell surface markers, we confirm that sEVs were present in all AH samples in UM patients. After brachytherapy, two subpopulations, CD63/81+ and CD9/63/81+ sEVs, constituted a significantly higher percentage of sEV distribution than before therapy. It is possible that CD63/81+ and CD9/63/81+ sEVs may be tumor-derived, although further studies are needed to identify the origin of these vesicles. We currently plan to expand our analysis of sEVs by using a multiplex bead-based flow cytometry assay with a panel that incorporates cancer-specific biomarkers. This approach would have the potential to improve our understanding of the origin of sEV subpopulations that are enriched after treatment.

## 4. Materials and Methods

This investigation was a case-series study at a tertiary care hospital (University of Southern California Roski Eye Institute). Samples were taken between August 2020 and May 2021.

### 4.1. Sample Collection

AH was collected from each patient before and after radioactive plaque placement, a form of localized radiation to treat UM. AH pre-brachytherapy samples were taken at the first surgery, before any significant radiation, and then again at the time of plaque removal, after a dose of 85 Gy to the tumor apex had been given (post-brachytherapy AH). As previously described in detail [29], clear corneal paracentesis was performed to extract up to 0.1 mL of AH by using a 32 gauge needle on a 1 cc syringe, as part of a routine procedure for anterior segment surgery at diagnosis or during treatment. Samples were transported on dry ice and stored at −80 °C until analysis.

### 4.2. Nanoparticle Tracking Analysis

By using 10 μL of unprocessed AH, NTA NanoSight NS300 Platform was used to evaluate the sizes and concentrations of extracellular vesicles and particles (EVPs). This platform is equipped with a 405 nm laser and sCMOS camera, which are used to record the Brownian movement of particles in suspension. The movement was then analyzed via the Stokes–Einstein equation to obtain the hydrodynamic radius and vesicle count for each modal size. NTA software 3.4 was used to perform data analysis, with the average of at least five camera recordings being presented. Results were shown as particle count per size distribution.

### 4.3. Single Particle-Interferometric Reflectance Imaging Sensor Analysis

Single sEV analysis was performed by using SP-IRIS-based ExoView R100 platform and ExoView Human Tetraspanin Kit (Unchained Labs, Pleasanton, CA, USA) as previously published [15,18]. Between 0.25 and 10 µL of unprocessed AH was diluted by using buffer A to a final volume of 40 µL. Then, 35 μL of each sample was incubated by using the ExoView Tetraspanin Chip at room temperature in an area free of vibrations or movement and sealed with tape to prevent drying out. The chips were then washed three times by using solution A from the kit and then incubated with immunocapture antibodies (anti-CD9 CF488, anti-CD81 CF555, and anti-CD63 CF647). Further information on antibodies can be found in the (Supplementary Material Figures S1 and S2). Antibodies were diluted as per the manufacturer’s protocol (Unchained Labs) to a final concentration of 0.5 μg/μL. After 1 h incubation at room temperature and subsequent washing and drying, the chips were then imaged with the ExoView R100 reader by using ExoView Scanner version 3.2 acquisition software; data were analyzed with ExoView Analyzer version 3.2. The volume of each sample loaded was calculated to ensure that particle counts fell within the instrument’s linear detection range (200 to 6000 particles per fluorescent channel). For the final analysis, particle counts were normalized to a standardized volume of 10 µL by using a dilution factor.

### 4.4. Statistical Analysis

Fisher’s exact test was used to compare categorical variables (sex, eye, eye color, ciliary body involvement, and PRAME Status; Table A1 and Table A2). Continuous variables were summarized as means ± standard error of mean (SEM) (all tables and figures). All continuous variables were non-normally distributed based on Shapiro–Wilk testing [30]. Non-normally distributed variables, such as EV counts and percentages, were compared by using non-parametric Wilcoxon Signed-Rank tests (paired samples; Figure 1, Figure 2 and Figure 3) and Mann–Whitney U tests (EV count comparison (Figure A1, Figure A2 and Figure A3) and EV subpopulation percentages across GLC versus UM (Figure A2)) [31]. Analysis of variance (ANOVA) testing (Kruskal–Wallis test) and Dunn’s multiple comparison tests were used for GEP classes and tumor locations (Figure A3). The ANOVA report can be found in the (Supplementary Material Table S1). Tetraspanin co-expression percentages were calculated based on the total number of fluorescent particles in the sample per SP-IRIS analysis. All statistical tests were 2-tailed, and *p* < 0.05 was considered statistically significant (Figure 1, Figure 2 and Figure 3 and Figure A1, Figure A2 and Figure A3; age, AJCC stage, and tumor stage in Table A1 and Table A2). Analyses were conducted and plots obtained by using Prism 10 (GraphPad, La Jolla, CA, USA).

## 5. Patents

Drs. Jesse L. Berry and Liya Xu have filed a patent application entitled Aqueous humor cell free DNA for diagnostic and Prognostic evaluation of Ophthalmic Disease.

## Data Availability

De-identified original datasets have been uploaded as Table A1 and Table A2.

## Supplementary Materials

The following supporting information can be downloaded at: https://www.mdpi.com/article/10.3390/ijms25116035/s1.

## Appendix A

**Table A1:** Univariate comparison of clinical characteristics between GEP1 and GEP2 UM patients.

Characteristic		GEP1, n = 12	GEP2, n = 4	*p*-Value
Sex (Fisher), n (%)				>0.999
	Females	7 (58.3)	3 (75.0)	
	Males	5 (41.7)	1 (25.0)	
Eye (Fisher), n (%)				0.569
	OD	7 (58.3)	1 (25.0)	
	OS	5 (41.7)	3 (75.0)	
Age at diagnosis, mean (±SD) (MWU)		53.3 (15.5)	58 (7.8)	0.425
Eye color (Fisher), n (%)				0.569
	Light (blue, gray, green, and hazel)	7 (58.3)	1 (25.0)	
	Dark (brown)	5 (41.7)	3 (75.0)	
Ciliary body involvement (Fisher), n (%)				>0.999
	Yes	6 (50.0)	2 (50.0)	
	No	6 (50.0)	2 (50.0)	
AJCC stage (linear-by-linear association), n (%)				0.009
	I	5 (41.7)	0 (0)	
	IIA	5 (41.7)	1 (25.0)	
	IIB	2 (16.6)	1 (25.0)	
	IIIA, IIIB, IIIC	0 (0)	2 (50.0)	
PRAME status, known in 17 cases (Fisher), n (%)				0.450
	Negative	11 (91.7)	3 (75.0)	
	Positive	1 (8.3)	1 (25.0)	
Tumor Stage (linear-by-linear association), n (%)				0.009
	T1a	5 (41.7)	0 (0)	
	T1b	4 (33.3)	0 (0)	
	T2a	1 (8.3)	1 (25.0)	
	T2b	1 (8.3)	1 (25.0)	
	T3	1 (8.3)	1 (25.0)	
	T4	0	1 (25.0)	

AJCC, American Joint Committee in Cancer; Fisher, Fisher’s exact test; GEP, gene expression profile; MWU, Mann–Whitney U test; OS, oculus sinister (left eye); OD, oculus dextrus (right eye); PRAME, preferentially expressed antigen in melanoma; SD, standard deviation.

**Table A2:** Comparison of clinical characteristics of UM patients with anterior and posterior tumors.

Characteristic		Posterior, n = 11	Anterior, n = 8	*p*-Value
Sex (Fisher), n (%)				0.633
	Females	6 (54.5)	6 (75.0)	
	Males	5 (45.4)	2 (25.0)	
Eye (Fisher), n (%)				0.370
	OD	4 (36.3)	5 (62.5)	
	OS	7 (63.7)	3 (37.5)	
Age at diagnosis, mean (±SD) (MWU)		57.2 (12.2)	54.0 (15.5)	0.888
Eye color (Fisher), n (%)				0.352
	Light (blue, gray, green, and hazel)	5 (45.4)	6 (75.0)	
	Dark (brown)	6 (54.5)	2 (25.0)	
GEP class (Fisher), n (%)				>0.999
	GEP1	6 (75.0)	6 (75.0)	
	GEP2	2 (25.0)	2 (25.0)	
AJCC stage (linear-by-linear association), n (%)				0.040
	I	8 (72.7)	0 (0)	
	IIA	1 (9.1)	5 (62.5)	
	IIB	1 (9.1)	2 (25.0)	
	IIIA, IIIB, IIIC	1 (9.1)	1 (12.5)	
PRAME status, known in 17 cases (Fisher), n (%)				>0.999
	Negative	7 (87.5)	7 (87.5)	
	Positive	1 (12.5)	1 (12.5)	
Tumor stage (linear-by-linear association), n (%)				0.011
	T1a	8 (72.7)	0 (0)	
	T1b	0 (0)	4 (50.0)	
	T2a	1 (9.1)	1 (12.5)	
	T2b	0 (0)	2 (25.0)	
	T3	1 (9.1)	1 (12.5)	
	T4	1 (9.1)	0 (0)	

AJCC, American Joint Committee in Cancer; Fisher, Fisher’s exact test; GEP, gene expression profile; MWU, Mann–Whitney U test; OS, oculus sinister (left eye); OD, oculus dextrus (right eye); PRAME, preferentially expressed antigen in melanoma; SD, standard deviation.
