# Supplementary material for: Comparative Single Vesicle Analysis of Aqueous Humor Extracellular Vesicles before and after Radiation in Uveal Melanoma Eyes
Source: Int J Mol Sci. 2024 May 30;25(11):6035. doi: 10.3390/ijms25116035 (PMC11172950; doi:10.3390/ijms25116035)
Supplement: Supplementary file 1 [file ijms-25-06035-s001.zip › Supplementary Figures S1 and S2.pdf]

Figure S1

Capture antibody information

Target	Clone	Description	Cross Reactivity
Human CD9	HI9a	Mouse, IgG1,κ	Bovine
Human CD81	JS-81	Mouse, IgG1,κ	
Human CD63	H5C6	Mouse, IgG1,κ	
Human CD41a	HIP8	Mouse, IgG1,κ	
Isotype control	MOPC-21	Mouse, IgG1,κ	

Figure S2

Detection antibody information

Target	Clone	Description	Cross Reactivity
Human CD9	HI9a	Mouse, IgG1,κ	Bovine
Human CD81	JS-81	Mouse, IgG1,κ	
Human CD63	H5C6	Mouse, IgG1,κ	
